# Supplementary material for: Characterization of enhancer activity in early human neurodevelopment using Massively Parallel Reporter Assay (MPRA) and forebrain organoids
Source: Sci Rep. 2024 Feb 16;14:3936. doi: 10.1038/s41598-024-54302-7 (PMC10873509; doi:10.1038/s41598-024-54302-7)
Supplement: Supplementary file 1 — Supplementary Information 1. [file 41598_2024_54302_MOESM1_ESM.pdf]

Title:
Characterization of enhancer activity in early human neurodevelopment using Massively Parallel Reporter Assay (MPRA) and forebrain organoids

Davide Capauto[1¶], Yifan Wang[2¶], Feinan Wu[1], Scott Norton[1], Jessica Mariani[1], Fumitaka Inoue[3], Gregory E. Crawford[4], The PsychENCODE Consortium[5], Nadav Ahituv[6,7], Alexej Abyzov[2]*, Flora M.Vaccarino[1,8,] *

¶ These authors contributed equally

[1] Child Study Center, Yale University, New Haven, CT 06520
[2] Department of Quantitative Health Sciences, Center for Individualized Medicine, Mayo Clinic, Rochester, MN 55905, USA
[3] Institute for the Advanced Study of Human Biology (WPI-ASHBi), Kyoto University; Kyoto, Japan
[4]Department of Pediatrics, Duke University, Durham, NC 27708 USA
[5]Consortium membership is listed in Supplemental Information.
[6]Department of Bioengineering and Therapeutic Sciences, University of California, San Francisco; San Francisco, CA, USA
[7]Institute for Human Genetics, University of California, San Francisco; San Francisco, CA, USA
[8]Department of Neuroscience, Yale University, New Haven, CT 06520, USA

*Co-corresponding authors. Email: abyzov.alexej@mayo.edu; flora.vaccarino@yale.edu

Supplemental Information

This supplement contains:

PsychENCODE Consortium membership list

Supplementary Figure 1 through 9

Supplemental References

Supplementary Tables 1through 5 are provided as separate Excel files.

Supplementary Table 1: **MPRA-tested enhancers activity across samples**. Enhancer: enhancer's genomic coordinates; label: NEGCTRL: negative control; POSCTRL: positive control; Number_samples_active: Number of samples with MPRA-active enhancers out of all 9 samples; Active: MPRA-active enhancers which pass the p-value threshold as described in Methods.

Supplementary Table 2: **Intersection of MPRA-tested enhancers with external datasets**. Active: MPRA-active enhancers which pass the p-value threshold as described in Methods;

iPSCs.active: Enhancers active in at least one sample at iPSC stage; TD0.active: Enhancers active in at least one sample at TD0; TD30.active: Enhancers active in at least one sample at TD30.

Supplementary Table 3: Enhancers activity, TFs and linked-genes expression. Enhancers coordinates, maximum activity at each time point, Transcription Factors predicted to bind with the tested sequences by FIMO, and enhancer subsets predicted by various criteria as well as linked genes as listed in the first tab.

Supplementary Table 4: Intersection of MPRA-tested enhancers with scATAC-seq.

Supplementary Table 5: Primers sequences for library amplification, virus titration and sequencing.

PsychENCODE Consortium list:

Schahram Akbarian[1], Alexej Abyzov[2], Nadav Ahituv[3], Dhivya Arasappan[4], Jose Juan Almagro Armenteros[5], Brian Beliveau[6], Jaroslav Bendl[1], Sabina Berretta[7], Rahul A. Bharadwaj[8], Arjun Bhattacharya[9], Lucy Bicks[9], Kristen Brennand[10], Davide Capauto[10], Frances A. Champagne[4], Tanima Chatterjee[10], Christos Chatzinakos[7], Yuhang Chen[10], Han-Chia Chen[11], Yuyan Cheng[9], Lijun Cheng[12], Andrew Chess[1], Jo-fan Chien[13], Zhiyuan Chu[10], Declan Clarke[10], Ashley Clement[3], Leonardo Collado-Torres[8], Gregory Cooper[14], Gregory Crawford[15], Rujia Dai[16], Nikolaos P. Daskalakis[7], Jose Davila-Velderrain[17], Amy Deep[8], Chengyu Deng[3], Chris DiPietro[7], Stella Dracheva[1], Shiron Drusinsky[18], Ziheng Duan[19], Duc Duong[20], Cagatay Dursun[10], Nick Eagles[8], Jonathan Edelstien[1],Prashant S. Emani[10], John Fullard[1], Kiki Galani[21], Timur Galeev[10], Michael J. Gandal[11], Sophia Gaynor[12], Mark Gerstein[10], Daniel Geschwind[9], Kiran Girdhar[1], Fernando S. Goes[22], William Greenleaf[5], Jennifer Grundman[9], Qiuyu Guo[9], Chirag Gupta[23], Yoav Hadas[1], Joachim Hallmayer[5], Xikun Han[21], Vahram Haroutunian[1], Natalie Hawken[9], Chuan He[24], Ella Henry[10], Joo Heon Shin[8], Stephanie Hicks[8], Marcus Ho[5], Li-Lun Ho[21], Gabriel E. Hoffman[1], Yiling Huang[5], Louise Huuki[8], Ahyeon Hwang[19], Thomas M. Hyde[8], Artemis Iatrou[7], Fumitaka Inoue[3], Aarti Jajoo[7], Matthew Jensen[10], Lihua Jiang[5], Peng Jin[20], Ting Jin[23], Connor Jops[11], Alexandre Jourdon[10], Riki Kawaguchi[9], Manolis Kellis[21], Joel Kleinman[8], Steven P. Kleopoulos[1], Alex Kozlenkov[1], Arnold Kriegstein[3], Anshul Kundaje[5], Soumya Kundu[5], Cheyu Lee ,University California Irvine[19], Donghoon Lee[1], Junhao Li[13], Mingfeng Li[10], Xiao Lin[1], Shuang Liu[10], Jason Liu[10], Jianyin Liu[9], Chunyu Liu[16], Shuang Liu[23], Shaoke Lou[10], Jacob Loupe[14], Dan Lu[25], Shaojie Ma[10], Liang Ma[26], Michael Margolis[9], Jessica Mariani[10], Keri Martinowich[8], Kristen R. Maynard[8], Samantha Mazariegos[9], Ran Meng[10], Richard Meyers[14], Courtney Micallef[1], Tatiana Mikhailova[16], Guo-li Ming[11], Shahin Mohammadi[27], Emma Monte[5], Kelsey S. Montgomery[25], Jill E. Moore[28], Jennifer Moran[12], Eran Mukamel[13], Angus Nairn[10], Charles Nemeroff[29], Pengyu Ni[10], Scott Norton[10], Tomasz Nowakowski[3], Larsson Omberg[25], Stephanie C. Page[8], Saejeong Park[10], Ashok Patowary[9], Reenal Pattni[5], Geo Pertea[8], Mette A. Peters[25], Nishigandha Phalke[28], Dalila Pinto[1], Milos Pjanic[1], Sirisha Pochareddy[10], Katherine

Pollard[18], Alex Pollen[3], Henry Pratt[28], Pawel F. Przytycki[18], Carolin Purmann[5], Zhaohui S. Qin[20], Ping-Ping Qu[5], Diana Quintero[9], Towfique Raj[1], Ananya S. Rajagopalan[10], Sarah Reach[1] , Thomas Reimonn[28], Kerry J. Ressler[7], Deanna Ross[4], Panagiotis Roussos[1], Joel Rozowsky[10], Misir Ruth[1], W. Brad Ruzicka[7], Stephan J. Sanders[30], Juliane M. Schneider[25], Soraya Scuderi[10], Robert Sebra[1], Nenad Sestan[10], Nicholas Seyfried[20], Zhiping Shao[1], Nicole Shedd[28], Annie W. Shieh[31], Mario Skarica[10], Clara Snijders[7], Hongjun Song[11], Matthew State[3], Jason Stein[32], Marilyn Steyert[3], Sivan Subburaju[7], Thomas Sudhof[5], Michael Snyder[5], Ran Tao[8], Karen Therrien[1], Li-Huei Tsai[21], Alexander Urban[5], Flora M. Vaccarino[10], Harm van Bakel[1], Daniel Vo[11], Georgios Voloudakis[1], Brie Wamsley[9], Tao Wang[5], Sidney H. Wang[31], Daifeng Wang[23], Yifan Wang[2], Jonathan Warrell[10], Yu Wei[16], Annika Weimer[5], Daniel R. Weinberger[8], Cindy Wen[9], Zhiping Weng[28], Sean Whalen[18], Kevin White[33], A Jeremy. Willsey[3], Hyejung Won[32], Wing Wong[5], Hao Wu[20], Feinan Wu[10], Stefan Wuchty[34], Dennis Wylie[4], Siwei Xu[19], Chloe X. Yap[34], Biao Zeng[1], Pan Zhang[9], Chunling Zhang[16], Bin Zhang[1], Jing Zhang[19], Yanqiong Zhang[32], Xiao Zhou[10], Ryan Ziffra[3], Trisha M. Zintel[25]

[1]Icahn School of Medicine at Mount Sinai, New York, NY, USA. [2]Mayo Clinic Rochester, Rochester, MN, USA. [3]University of California, San Francisco, San Francisco, CA, USA. [4]The University of Texas at Austin, Austin, TX, USA. [5]Stanford University, Stanford, CA, USA. [6]University of Washington, Seattle, WA, USA. [7]McLean Hospital, Belmont, MA, USA. [8]Lieber Institute for Brain Development, Baltimore, MD, USA. [9]University of California, Los Angeles, Los Angeles, CA, USA. [10]Yale University, New Haven, CT, USA. [11]University of Pennsylvania, Philadelphia, PA, USA. [12]Tempus Labs, Inc., Chicago, IL, USA. [13]University of California, San Diego, San Diego, CA, USA. [14]HudsonAlpha Institute for Biotechnology, Huntsville, AL, USA. [15]Duke University, Durham, NC, USA. [16]SUNY Upstate Medical University, Syracuse, NY, USA. [17]Human Technopole, Milan, Italy. [18]Gladstone Institutes, University of California, San Francisco, San Francisco, CA, USA. [19]University of California, Irvine, Irvine, CA, USA. [20]Emory University, Atlanta, GA, USA. [21]Massachusetts Institute of Technology, Cambridge, MA, USA. [22]Johns Hopkins University, Baltimore, MD, USA. [23]University of Wisconsin-Madison, Madison, WI, USA. [24]The University of Chicago, Chicago, IL, USA. [25]Sage Bionetworks, Seattle, WA, USA. [26]The University of Texas Health Science Center at San Antonio, San Antonio, TX, USA. [27]Broad Institute of MIT and Harvard, Cambridge, MA, USA. [28]University of Massachusetts Chan Medical School, Worcester, MA, USA. [29]The University of Texas at Austin Dell Medical School, Austin, MA, USA. [30]University of Oxford, Oxford, England, UK. [31]The University of Texas Health Science Center at Houston, Houston, TX, USA. [32]University of North Carolina at Chapel Hill, Chapel Hill, USA. [33]National University of Singapore, Singapore, Singapore. [34]University of Miami, Miami, FL, USA. University of Queensland, Queensland, NZ.

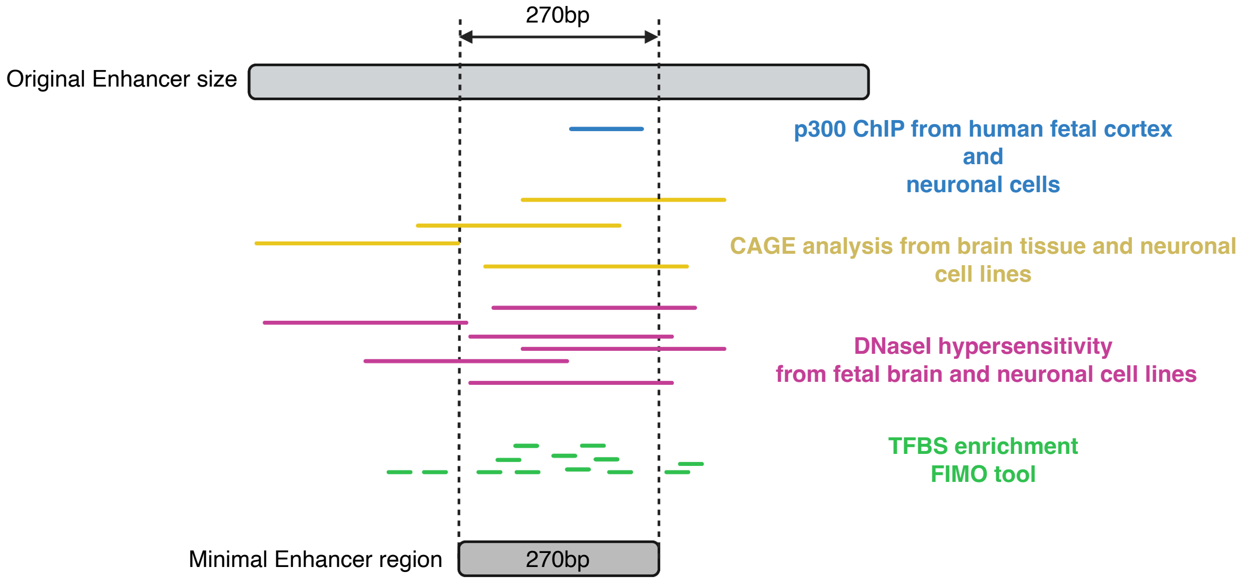

Supplementary Figure S1. Minimal enhancer region selection.
We selected a minimal enhancer region of 270bp by intersecting the enhancer coordinates with external datasets, and then further refined the region using FIMO based on the highest number of TFBSs. External datasets: i) p300 ChIP-seq peaks from human neuronal cell lines [1] and human fetal cortex [2], ii) Cap analysis of gene expression (CAGE) analysis from brain tissues and neuronal cell types [3], and iii) DNase hypersensitivity peaks from neuronal progenitor cells and brain tissue [4].

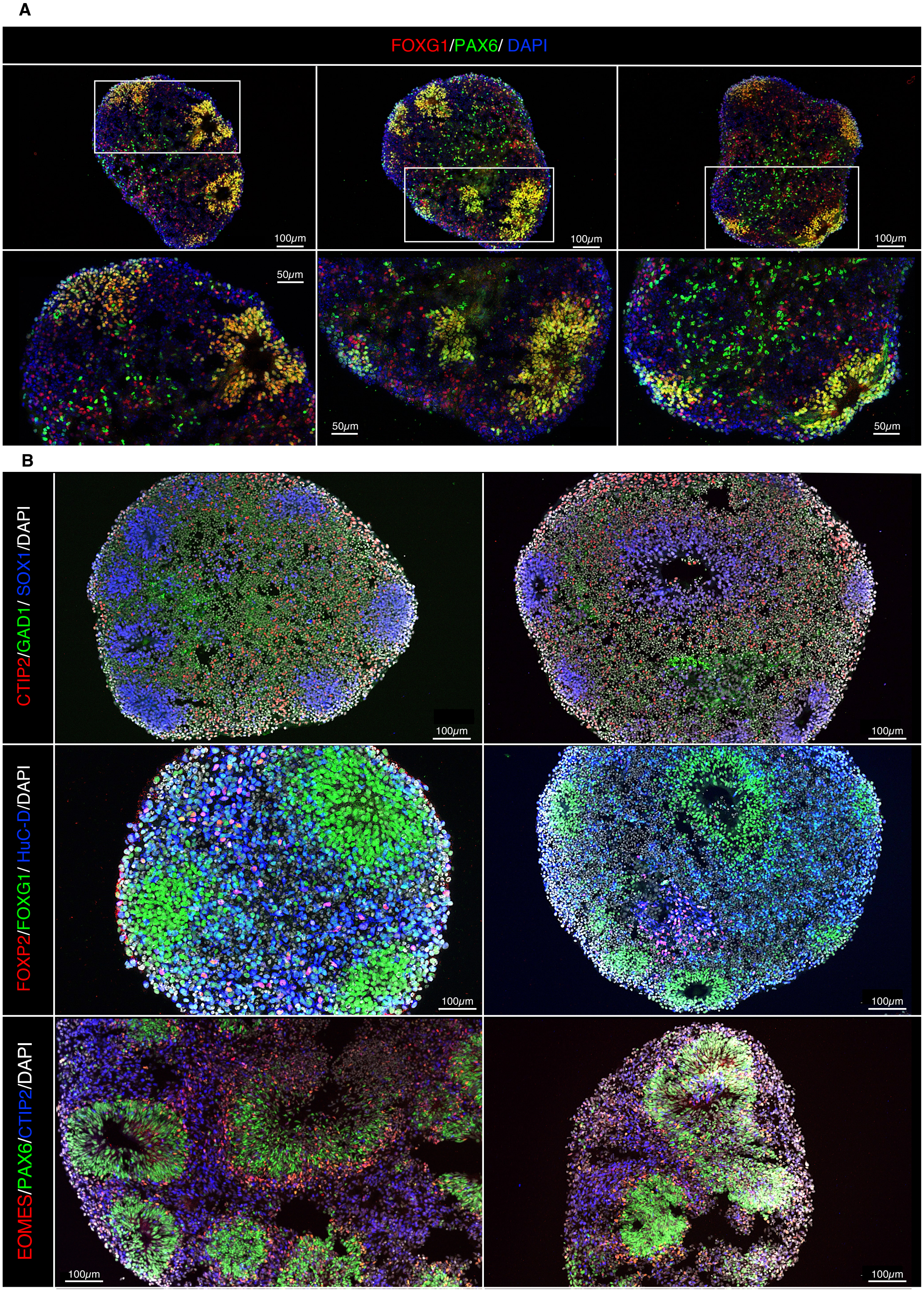
A
FOXG1/PAX6/ DAPI
100μm
100μm
100μm
50μm
50μm
50μm
B
CTIP2/GAD1/ SOX1/DAPI
100μm
100μm
FOXP2/FOXG1/ HuC-D/DAPI
100μm
100μm
EOMES/PAX6/CTIP2/DAPI
100μm
100μm

Supplementary Figure S2. Immunocytochemical characterization of forebrain organoids. Representative immunostaining of forebrain organoids at TD30.

(A) PAX6+ and FOXG1+ immunostaining showing progenitor cells clustered in “rosettes” demonstrating telencephalic progenitor fate. **(B)** Immunostaining for more mature neuronal cells outside of rosettes, such as FOXP2+ and CTIP2+ excitatory neurons of layer 5-6 cerebral cortex, GAD1+ inhibitory neurons and young HuC-D+ neurons. Immunostaining for SOX1 and EOMES shows proliferating and intermediate cortical progenitor cells, respectively.

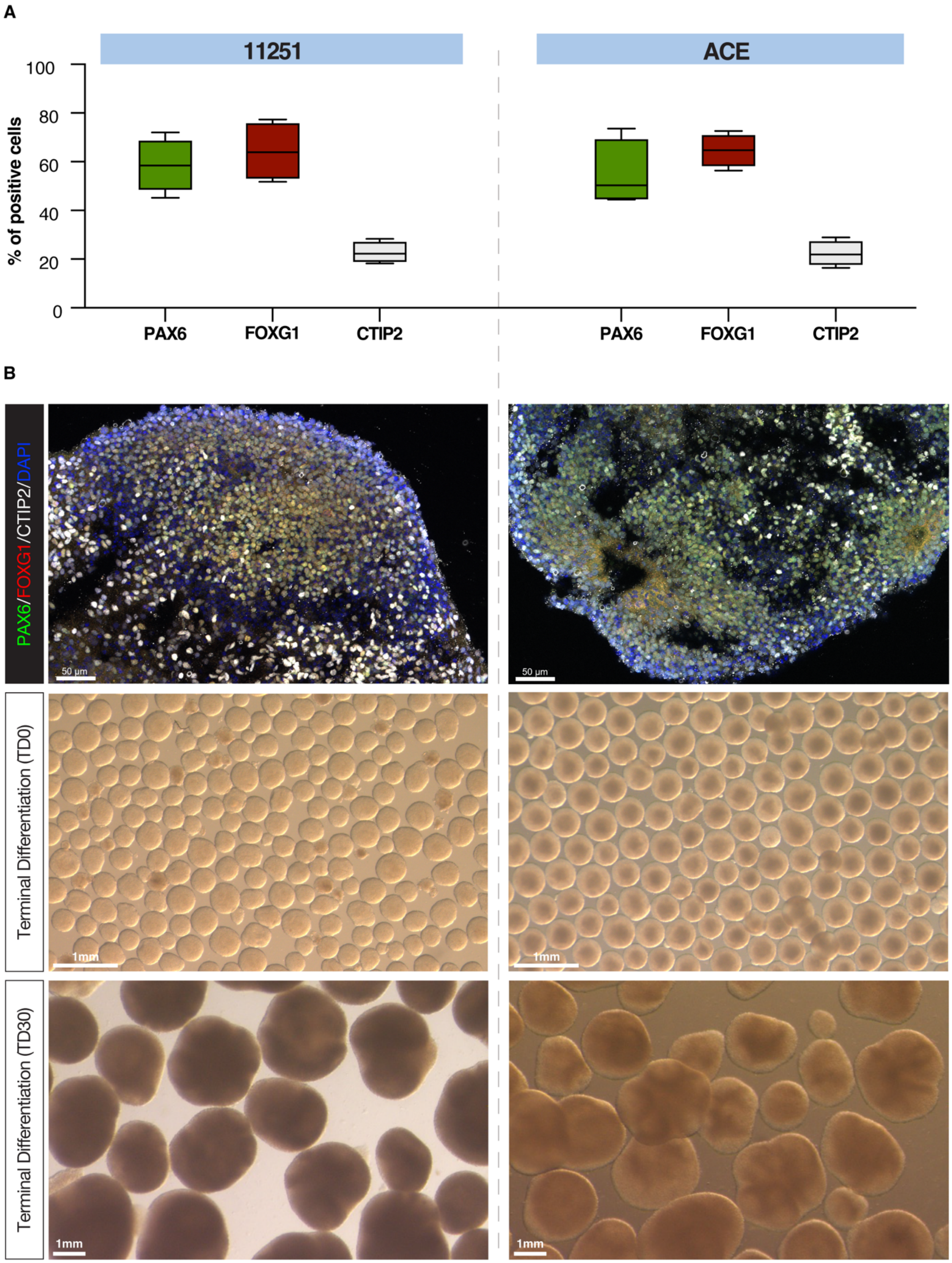

A
11251
ACE
100
80
60
40
20
0
% of positive cells
PAX6
FOXG1
CTIP2
PAX6
FOXG1
CTIP2
B
PAX6/FOXG1/CTIP2/DAPI
50 µm
50 µm
Terminal Differentiation (TD0)
1mm
1mm
Terminal Differentiation (TD30)
1mm
1mm

Supplementary Figure S3. Immunocytochemistry for PAX6, FOXG1, CTIP2 in forebrain organoids.

(A) Box and whisker plots (including minima and maxima) showing immunocytochemical quantification of PAX6-, FOXG1- and CTIP2-positive cells in TD30 organoids of the 11251 and ACE iPSC lines used for the MPRA experiment (the ACE line used as technical replicate); n = 4 organoids per sample. The Tukey method was used to plot boxes and whiskers (minima, maxima); mean value is shown as center line and it is calculated across the four organoids of each line. **(B)** Upper panels: representative images of PAX6, FOXG1 and CTIP2 immunostainings of organoids at TD30. Scale Bar: 50 µm. Middle and lower panels: representative bright field images of 11251 and ACE cortical organoids at TD0 and TD30 using a 2X and 4X objective respectively. Scale Bar: 1mm.

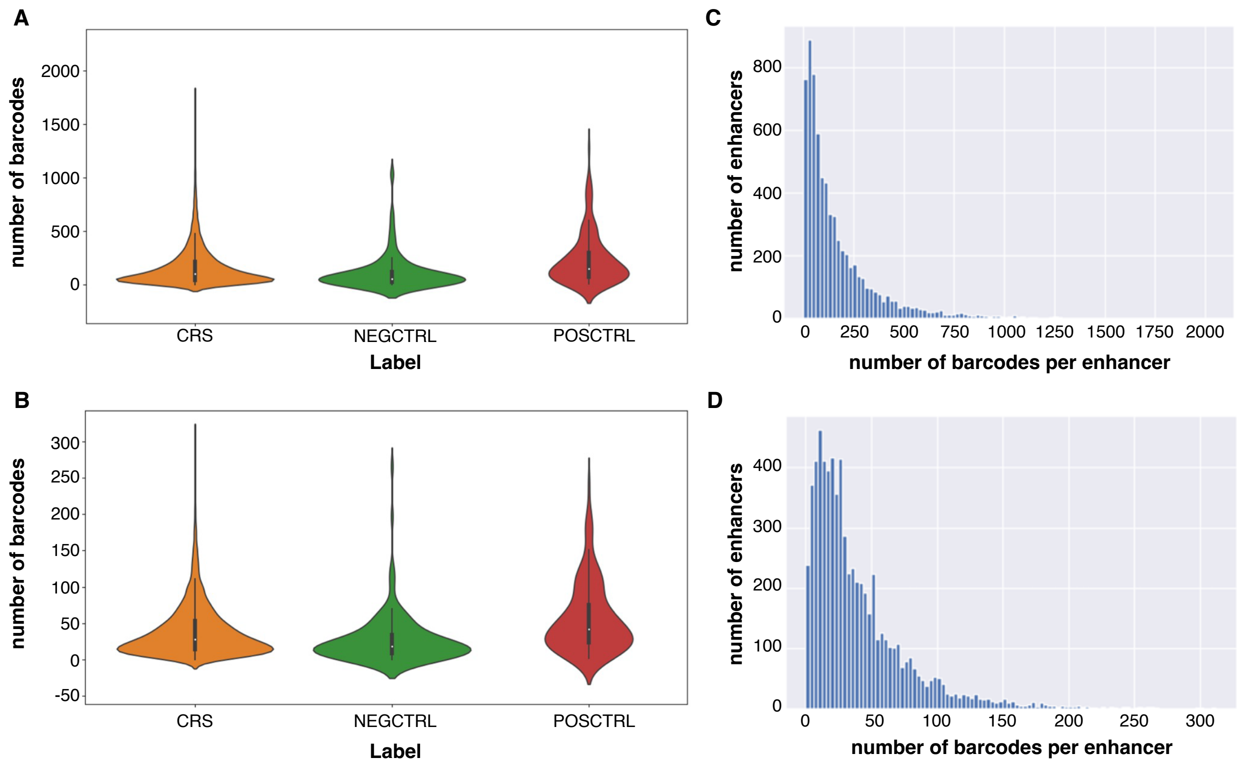

Supplementary Figure S4. Statistics of MiSeq barcodes for tested enhancers. (A) Number of barcodes per enhancer before quality filtering in candidate regulatory sequences (CRS), positive controls (POSCTRL) and negative controls (NEGCTRL). **(B)** Number of barcodes per enhancer after quality filtering in CRS, POSCTRL and NEGCTRL. **(C)** Distribution of number of barcodes per enhancer before filtering. **(D)** Distribution of number of barcodes per enhancer after filtering.

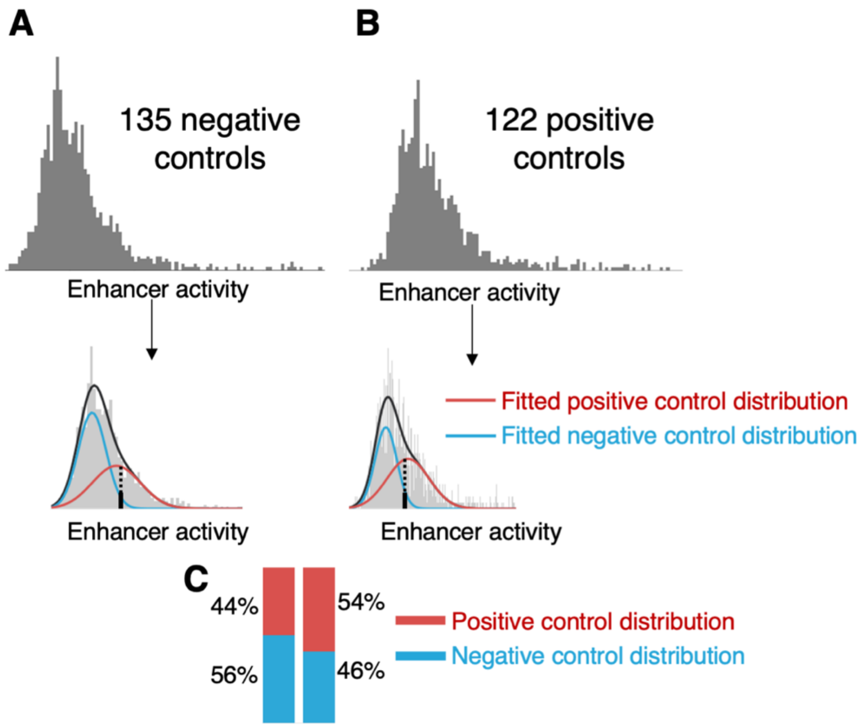

Supplementary Figure S5. Gaussian mixture model with positive and negative controls. **(A)** Gaussian mixture model fitted using negative reference data. **(B)** Gaussian mixture model fitted using positive reference data with the same parameter as from negative controls, but with different ratio of negative and positive control distributions. **(C)** Ratio of negative control distributions and positive control distributions in positive and negative control data. The majority of positive control data (right) consists of positive control distribution, while the majority of negative control data (left) consists of negative control distribution.

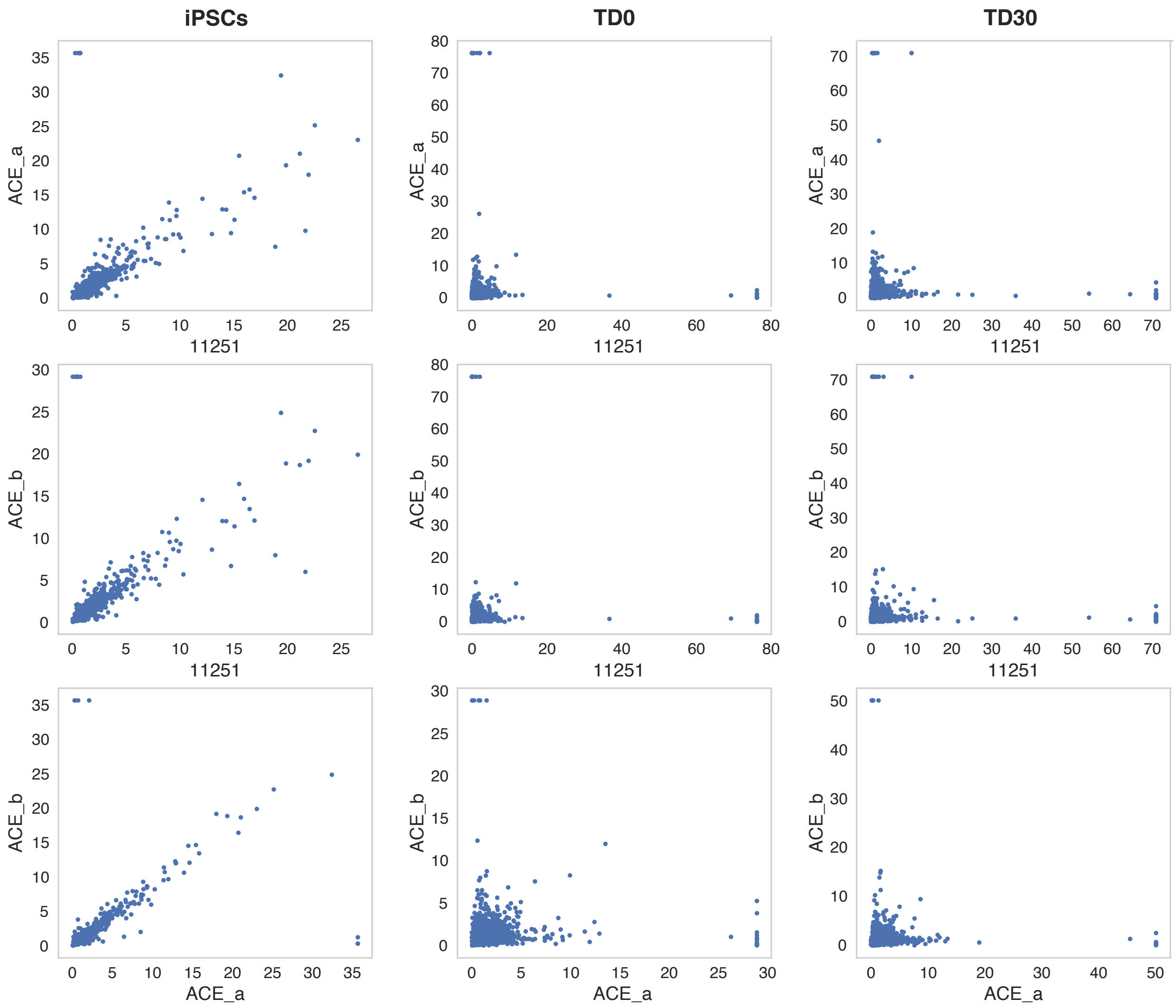

Supplementary Figure S6. RNA/DNA ratios in all tested enhancers at iPSC, TD0 and TD30 comparing between different samples.

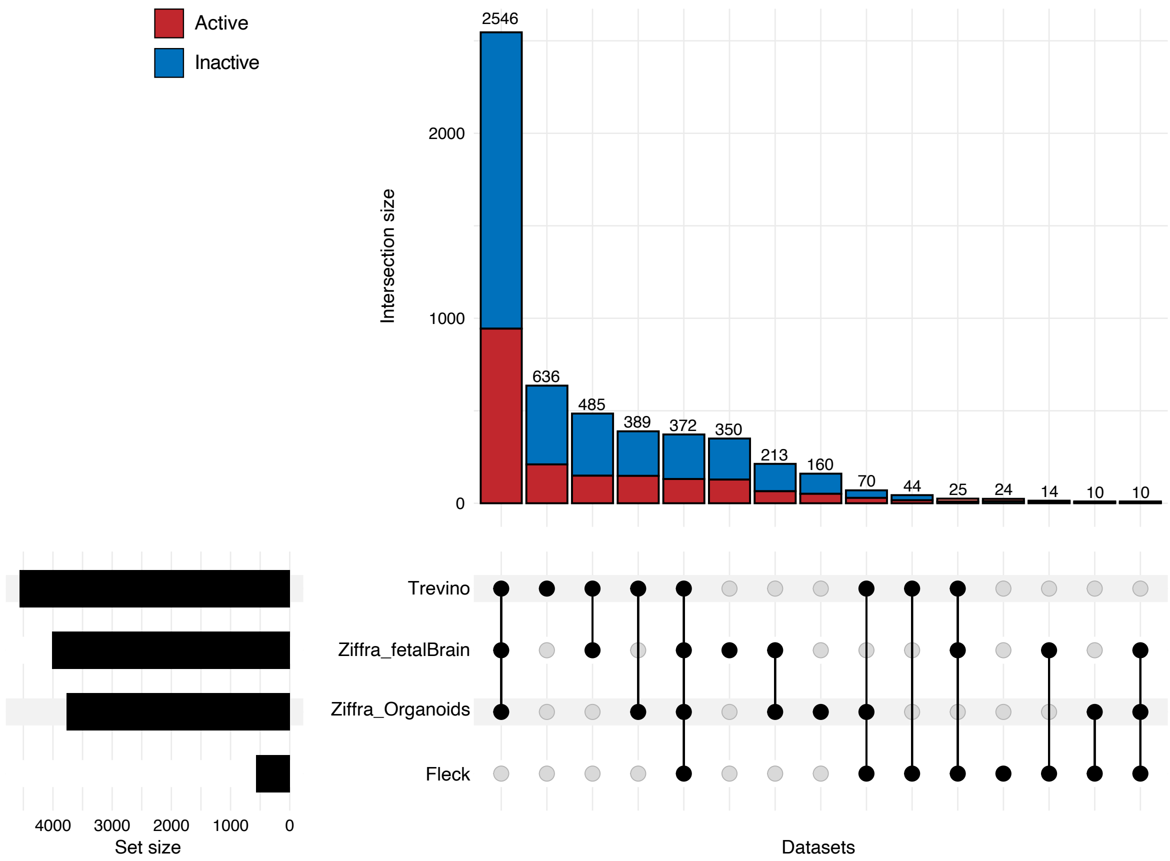

Supplementary Figure S7. Intersection with external datasets

Upset plot showing the number of intersections between active (red) and inactive (blue) enhancers and external datasets [5, 6]. Compared to the Upset plot in Fig. 2B, the Ziffra scATAC-seq dataset is separated into two groups: fetal brain and organoids.

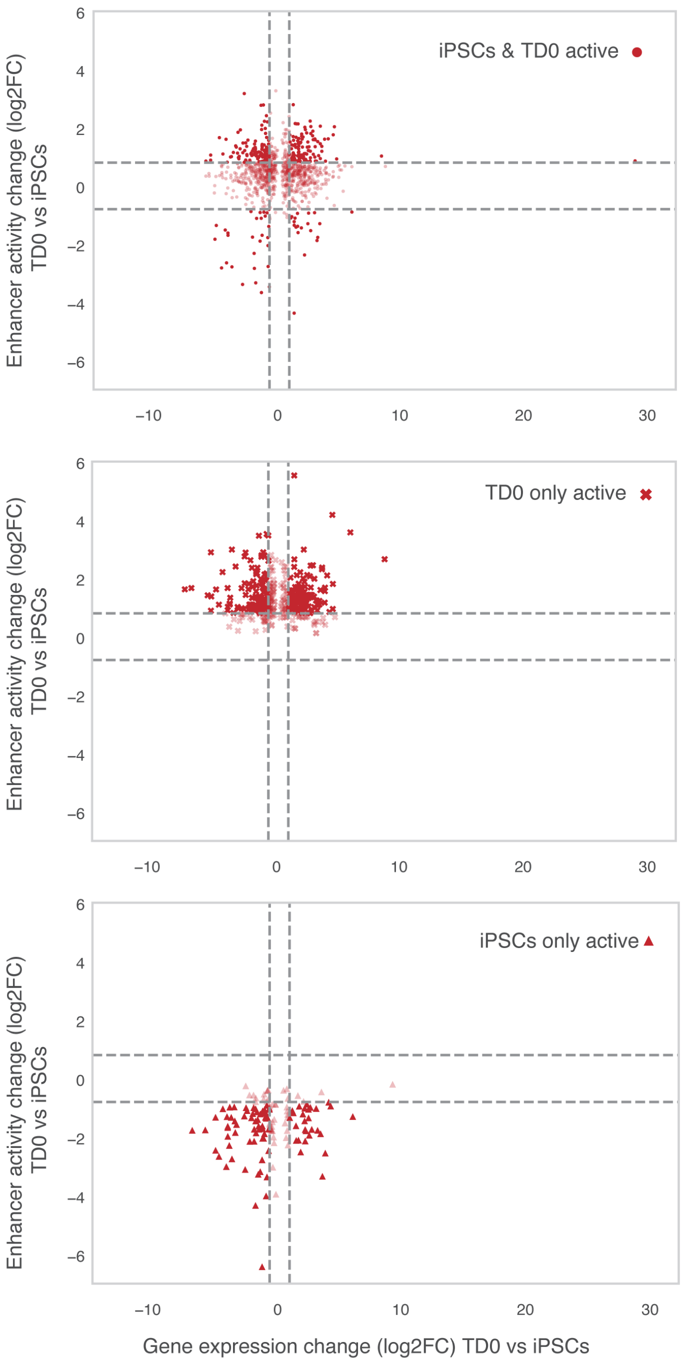

iPSCs & TD0 active
TD0 only active
iPSCs only active
Enhancer activity change (log2FC) TD0 vs iPSCs
Gene expression change (log2FC) TD0 vs iPSCs

Supplementary Figure S8. MPRA-active enhancers positively or negatively correlate with the expression of the corresponding linked genes. Scatter plots of change in MPRA activity for an enhancer (y-axis) and expression of a linked gene in the native genomic context. The X and Y axes represent differences in, respectively, enhancer activity and gene expression between TD0 and iPSC. Each dot represents an enhancer and linked gene pair. Circles (top) represent enhancers identified as active at both iPSC and TD0; crosses (middle) represent enhancers identified as active only at TD0; triangles represent enhancers identified as active only in iPSC.

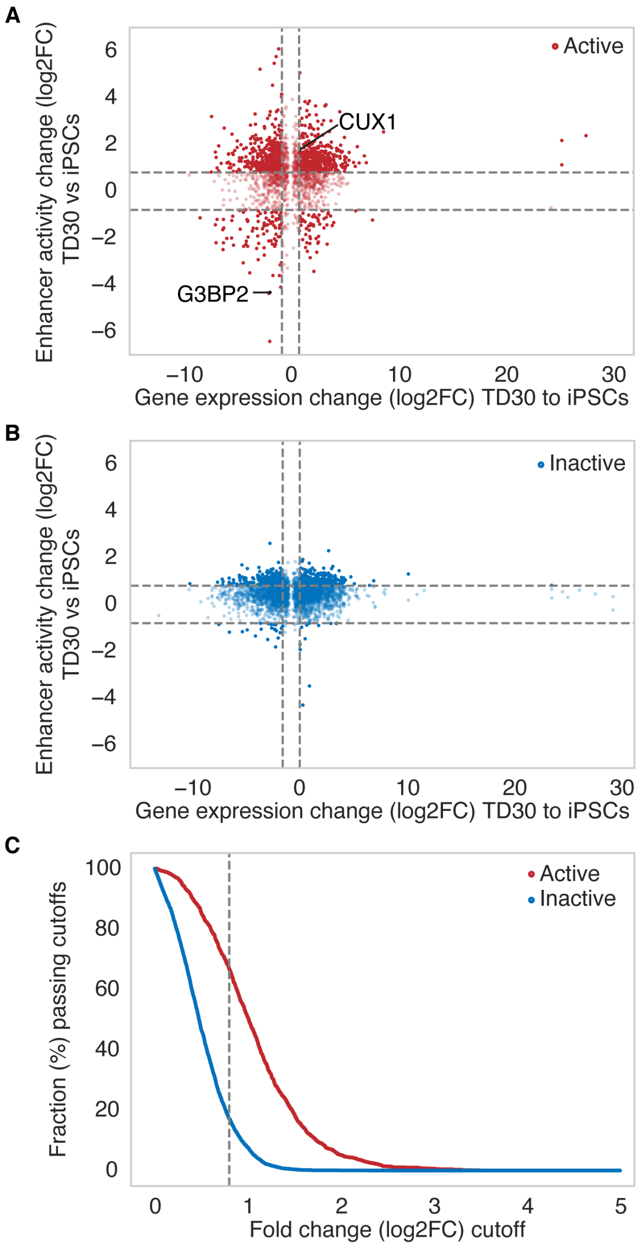

Supplementary Figure S9. MPRA-active enhancers correlate with differences in expression of linked genes across timepoints. (A) and (B) Scatter plots of change in MPRA activity for an enhancer (y-axis) and change of expression for a linked gene. The X and Y axes represent differences in, respectively, enhancer activity and gene expression between TD30 and iPSC. Each dot represents a pair of an enhancer and a linked gene. iPSC or TD30 active enhancers are shown as red circles; inactive enhancers are represented as blue circles. Dotted lines represent confidence interval cutoff. **(C)** More correlated enhancer-gene pairs are observed in active enhancers compared to inactive enhancers. The Y-axis represents the percentage of enhancer-gene pair passing the log2 fold change cutoff for enhancer activity and linked gene expression. The X-axis represents the log2 fold change gene expression cutoff. The dashed line represents cutoff at log2FC=0.8, which is the same as marked in panel **(A)**.

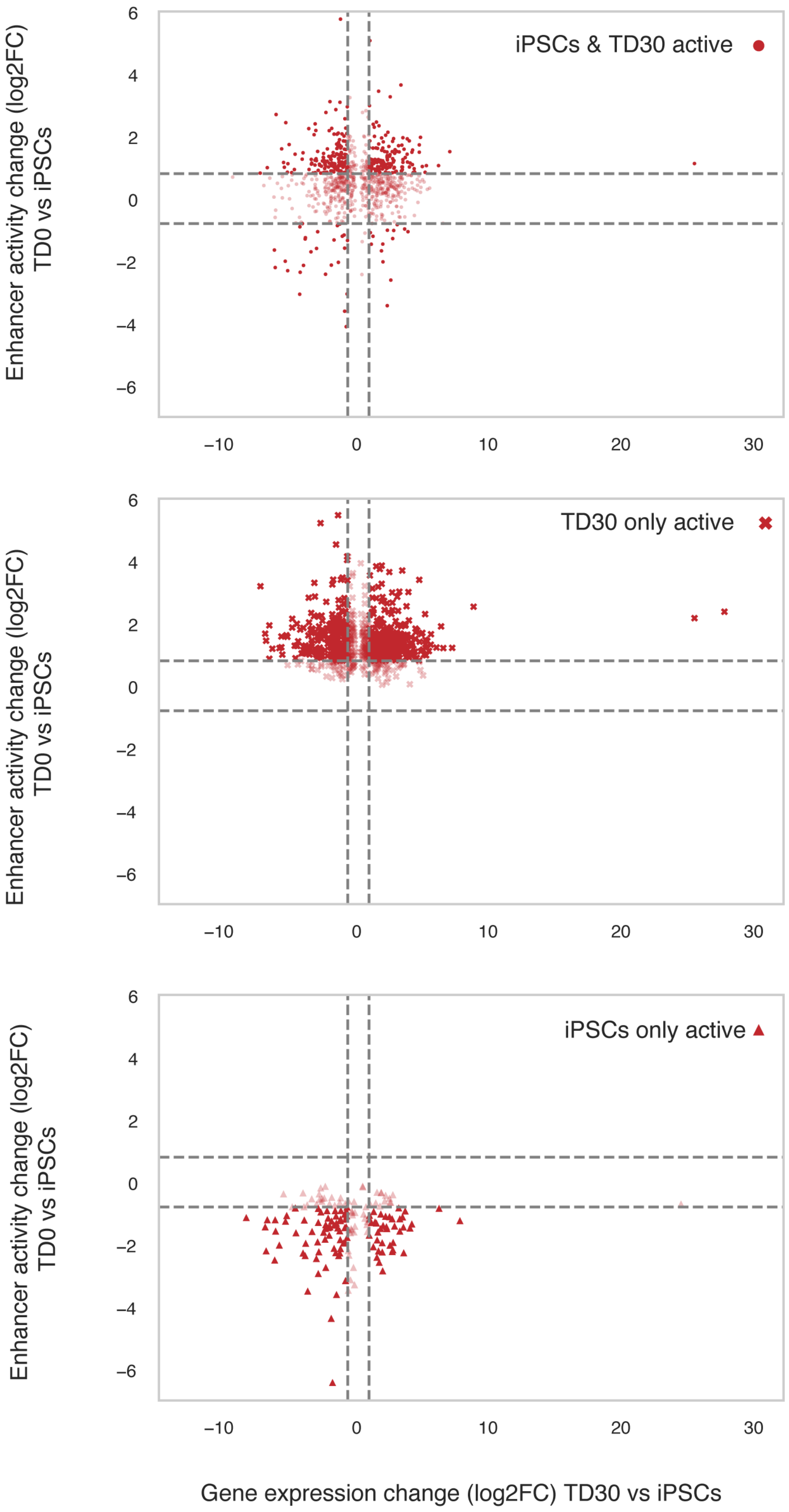
iPSCs & TD30 active
TD30 only active
iPSCs only active
Enhancer activity change (log2FC) TD0 vs iPSCs
Gene expression change (log2FC) TD30 vs iPSCs
6
4
2
0
−2
−4
−6
−10
0
10
20
30

Supplementary Figure S10. MPRA-active enhancers positively or negatively correlate with expression of the corresponding linked genes. Scatter plots of change in MPRA activity for an enhancer (y-axis) and expression for a linked gene in the native genomic context. The X and Y axes represent differences in, respectively, enhancer activity and gene expression between TD30 and iPSC. Each dot represents an enhancer and a linked gene pair. Circles (top) represent enhancers identified as active at both iPSC and TD30; crosses (middle) represent enhancers identified as active only at TD30; triangles represent enhancers identified as active only in iPSC.

Supplemental References

1. Consortium EP. An integrated encyclopedia of DNA elements in the human genome. Nature. 2012;489(7414):57-74. Epub 2012/09/08. doi: 10.1038/nature11247. PubMed PMID: 22955616; PubMed Central PMCID: PMCPMC3439153.
2. Visel A, Taher L, Girgis H, May D, Golonzhka O, Hoch RV, et al. A high-resolution enhancer atlas of the developing telencephalon. Cell. 2013;152(4):895-908. Epub 20130131. doi: 10.1016/j.cell.2012.12.041. PubMed PMID: 23375746; PubMed Central PMCID: PMCPMC3660042.
3. Andersson R, Gebhard C, Miguel-Escalada I, Hoof I, Bornholdt J, Boyd M, et al. An atlas of active enhancers across human cell types and tissues. Nature. 2014;507(7493):455-61. doi: 10.1038/nature12787. PubMed PMID: 24670763; PubMed Central PMCID: PMCPMC5215096.
4. Meuleman W, Muratov A, Rynes E, Halow J, Lee K, Bates D, et al. Index and biological spectrum of human DNase I hypersensitive sites. Nature. 2020;584(7820):244-51. Epub 20200729. doi: 10.1038/s41586-020-2559-3. PubMed PMID: 32728217; PubMed Central PMCID: PMCPMC7422677.
5. Trevino AE, Sinnott-Armstrong N, Andersen J, Yoon SJ, Huber N, Pritchard JK, et al. Chromatin accessibility dynamics in a model of human forebrain development. Science. 2020;367(6476). doi: 10.1126/science.aay1645. PubMed PMID: 31974223; PubMed Central PMCID: PMCPMC7313757.
6. Ziffra RS, Kim CN, Ross JM, Wilfert A, Turner TN, Haeussler M, et al. Single-cell epigenomics reveals mechanisms of human cortical development. Nature. 2021;598(7879):205-13. Epub 2021/10/08. doi: 10.1038/s41586-021-03209-8. PubMed PMID: 34616060; PubMed Central PMCID: PMCPMC8494642.
